# Tuba ovarian abscesses formation from decidualized ovarian endometrioma after appendiceal endometriosis presenting as acute appendicitis in pregnancy 

**Published:** 2012-05

**Authors:** Erbil Dogan, Emre Okyay, Bahadir Saatli, Safak Olgan, Sulen Sarioglu, Meral Koyuncuoglu

**Affiliations:** 1 *Department of Obstetrics and Gynecology, Dokuz Eylul University, School of Medicine, Izmir, Turkey.*; 2 *Department of Pathology, Dokuz Eylul University, School of Medicine, Izmir, Turkey.*

**Keywords:** *Endometriosis*, *Deciduosis*, *Acute appendicitis*, *Tuba ovarian abscess*

## Abstract

**Background:** Acute appendicitis with appendicial endometriosis is a very infrequently encountered condition during pregnancy. Decidualization is the hypertrophy of endometrial stromal cells by the effect of progesterone. Similarly, in pregnancy, ectopic stromal endometrial cells in endometriosis can also be transformed by the same mechanism and ectopic decidua (deciduosis) may occur.

**Case:** Here we report a 30 year old pregnant woman presenting twice with acute abdominal symptoms requiring surgery for appendicial and ovarian endometriosis and deciduosis. We emphasize that deciudualized endometriosis may first present during pregnancy with acute abdomen necessitating emergency laparotomy and complicating the course of gestation.

**Conclusion:** To our knowledge only 9 cases in which decidualized endometriotic tissue causing acute abdomen necessitating surgery during pregnancy were reported in the literature. What makes our case special is that the patient needed two laparotomies during the pregnancy period which was a very stressful situation for both the patient and the physicians.

## Introduction

Acute appendicitis with appendicial endometriosis is a very infrequently encountered condition during pregnancy which ranges between 3-8 deliveries per 10,000 (1). Decidualization is the hypertrophy of endometrial stromal cells by the effect of progesterone. Similarly, in pregnancy, ectopic stromal endometrial cells in endometriosis can also be transformed by the same mechanism and ectopic decidua (deciduosis) may occur (2). 

Here we report a 30 year old pregnant woman presenting twice with acute abdominal symptoms requiring surgery for appendicial and ovarian endometriosis and deciduosis.

## Case Report

A 30-year old, gravida 1, para 0, woman at 24th week of pregnancy presented with right lower quadrant pain, fever and nausea. Axillary body temperature and pulse rate were 37.3^o^C and 104/min, respectively. Abdominal examination revealed tenderness, guarding, and rebound tenderness at the right side of the uterus. White blood cell count was 19,500 cells/mm^3^ with 92.8% neutrophils. 

The sonographic examination did not reveal any findings consistent with acute appendicitis exception for periceaceal fluid and it showed a normal intrauterine pregnancy. With a preoperative diagnosis of acute appendicitis, a laparotomy was done via a paramedian incision in the right side of the abdomen. Appendix was erythematous and appendicectomy was performed. 

The histopathological examination of the appendix revealed acute appendicitis with extensive deciduosis of the appendiceal wall of all the three layers. There were also glandular structures among the decidua which were positive with cytokeratin 7, while the adjacent appendicial mucosa was negative. The diagnosis was endometriosis and decidiosis of appendix and acute appendicitis. The patient was discharged with antibioteraphy without further complaints. 

One month later, at 28 weeks’ pregnancy, the patient came again with left lower quadrant pain, high fever and vomiting. Her temperature and pulse rate were 38.5^o^C and 110/min, respectively. At physical examination, the patient had 7 months of pregnant uterus and acute abdominal findings consisting of mainly left lower quadrant tenderness. Laboratory results showed a white blood cell count of 23, 800 cells/mm^3^. A pelvic sonogram revealed a normal intrauterine pregnancy at 28 weeks and free fluid between the intestinal structures and a 5 cm complicated left ovarian cystic mass compatible with tubo-ovarian abscess. At laparotomy with a median subumbilical incision, a small amount of purulent free fluid and extensive pelvic deciduosis covering all the pelvic peritoneal surfaces with left tuboovarian abscess was observed. The abscess was drained and left salpingectomy was performed. 

Papillary excrescences were observed protruding into the lumen of the mass and ovarian biopsies were obtained. The abdominal cavity was washed with warm saline solution and a drain was placed in the Douglas pouch for drainage which was removed 3 days later. Early postoperative recovery was uneventful but on the 5^th ^postoperative day the patient had uterine contractions and tocolytic therapy was initiated with nifedipine. She gave birth to a 1400 gr healthy male baby with spontaneous vaginal delivery. 

Histo-pathological examination revealed ovarian endometriosis with marked decidual changes and hemosiderin-laden macrophages as well as moderate amount of polynuclear leukocytosis in cyst wall. The final pathological report confirmed infected endometrioma with deciduosis. Postoperatively patient received tazocin antibiotherapy for 3 weeks and discharged from the hospital without further complications. At the second postpartum month she was completely recovered and her baby was doing well without any complications related to prematurity.

**Figure 1:**
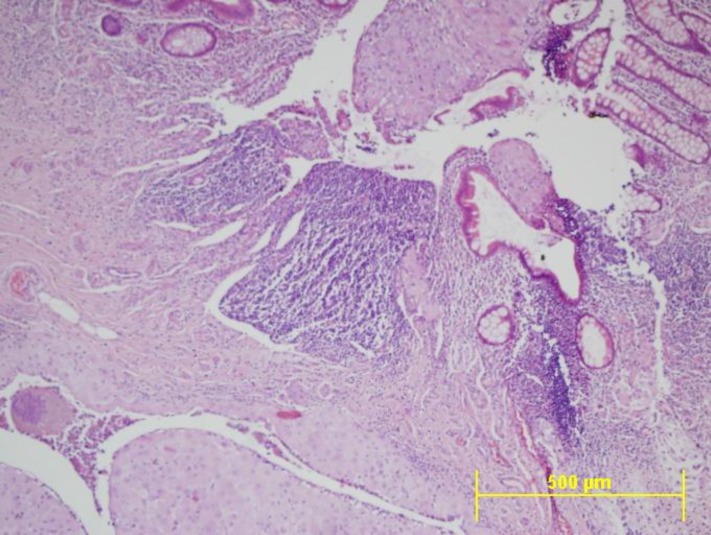
Extensive deciduosis of the appendiceal wall including mucosal, muscular and serosal layers. Note ulceration of the appendiceal mucosa and intraluminal decidual tissue. Also the glandular structures can be identified at the decidual submucosal area (H&E, original magnification X10).

## Discussion

Endometriosis of appendix is a rarely encountered condition, occurring with the rate of 0.2-0.3% in appendectomies (3). Recently, its prevalence was reported to be 2.8% and it is noted that when pelvic endometriosis is present, odds ratio for presence of appendiceal endometriosis was 20.9 compared with general population (4). There were no studies established which explained mechanism of appendiceal involvement. Although it is usually asymptomatic, some cases may present with perforation, intussusception and mucocel (5). 

Therefore, endometriosis of the appendix is often an incidental finding during abdominal operations. Deciduosis appendix is encountered during pregnancy and it is frequently identified at the serosal region of the appendix. At this localization the decidualisation was attributed to the physiological reaction of the pluripotential submesothelial stromal cells to hormonal influences of the pregnancy (6). In some cases, the desidualisation was associated with evident endometrial glands as in our case which could also be visualized by demonstration of cytokeratin 7 by immunohistochemistry (Figure 1) (1).

Adnexal masses complicating pregnancy have been reported to occur in average 1 in 600 of live births and approximately 11% of these are endometriomas (7). These decidual changes of endometriosis can be characterized by mural nodules and that macroscopically mimics a malignant tumor. Therefore, in literature, there are several reported cases and all of them have resulted in surgery during pregnancy because of the suspicious imaging findings (2). Separately, there is one reported case that had an infected endometrioma during pregnancy. 

In conclusion, we emphasize that deciudualized endometriosis may first present during pregnancy with acute abdomen necessitating emergency laparotomy and complicating the course of gestation. To our knowledge only 9 cases in which decidualized endometriotic tissue causing acute abdomen necessitating surgery during pregnancy were reported in the literature (8). 

What makes our case special is that the patient needed two laparotomies during the pregnancy period which was a very stressful situation for both the patient and the physicians.
